# Effect of Trendelenburg’s Operation With Subfascial Ligation of Perforators in Clinical Improvement and Quality of Life Among Patients With Varicose Veins

**DOI:** 10.7759/cureus.41472

**Published:** 2023-07-06

**Authors:** Sri Hari Priya Vemulakonda, Uday Kumbhar, Sagar Prakash, Oseen Shaikh, Gopal Balasubramanian, Chellappa Vijayakumar, Abhinaya Reddy, Suresh Chilaka, Muhsina Kunjumohammed, Bhavana Katta

**Affiliations:** 1 Surgery, Jawaharlal Institute of Postgraduate Medical Education and Research, Puducherry, IND

**Keywords:** chronic venous disease, venous insufficiency, quality of life, trendelenburg’s surgery, varicose veins

## Abstract

Background

Assessing patients’ quality of life has received increasing attention, mainly because questions have been raised regarding the direct benefits of the treatment provided. Hence, clinical outcomes and quality of life must be measured after chronic venous disease treatment. The primary objective of the study was to assess the improvement in clinical outcome and improvement in quality of life using the revised venous clinical severity score and chronic venous insufficiency questionnaire-14, respectively, in patients with varicose veins undergoing Trendelenburg’s surgery and subfascial ligation of perforators. The secondary objective was to identify the relationship between the revised venous clinical severity score and the chronic venous insufficiency questionnaire-14 score.

Method

The present study is a single-center, prospective cohort study to assess the clinical improvement and quality of life in patients with varicose veins undergoing Trendelenburg surgery and subfascial ligation of perforators. All the study participants were evaluated preoperatively with the clinical, etiological, anatomical, and pathophysiological stage of the disease, revised venous clinical severity score for the clinical severity, and the chronic venous insufficiency questionnaire-14 questionnaire for the quality of life. The study participants were reviewed 90 days after surgery and reassessed for clinical severity and quality of life, both scores.

Results

Of the 87 screened varicose vein patients, 52 were included in the study. However, one patient was lost to follow-up. There were 38 (74.5%) males and 13 (25.5%) females. There was a significant difference in the preoperative and postoperative mean revised venous clinical severity score of the C3, C4, and C6 stages of the disease (p-value = <0.01). There was a significant difference in the mean preoperative and postoperative chronic venous insufficiency questionnaire-14 score in C3, C4, and C6 (p-value = <0.01). There was a significant difference in the median preoperative and postoperative revised venous clinical severity score (p-value = <0.01). There was a considerable difference in the mean preoperative and postoperative chronic venous insufficiency questionnaire-14 score (p-value = <0.01). The correlation coefficient between the preoperative chronic venous insufficiency questionnaire-14 score and the revised venous clinical severity score was 0.26 (p-value = 0.58), and the correlation coefficient between the postoperative chronic venous insufficiency questionnaire-14 score and the revised venous clinical severity score was 0.42 (p-value = <0.01).

Conclusion

Patients undergoing Trendelenburg’s surgery and subfascial ligation of perforators for varicose veins significantly improved the clinical severity and quality of life. There was significant improvement among the overall revised venous clinical severity score and chronic venous insufficiency questionnaire-14 score after surgery among the different clinical classes. There was no preoperative correlation between the revised venous clinical severity score and the chronic venous insufficiency questionnaire-14 score. However, there was a significant correlation between the postoperative revised venous clinical severity score and chronic venous insufficiency questionnaire-14 score.

## Introduction

Varicose veins (VV) are abnormally dilated tortuous superficial veins that can occur due to anatomical abnormalities, congenital syndromes, or primary or secondary insufficiency. VV surgery is a standard, elective general surgical procedure. There is still an ambiguity among surgeons regarding the treatment of VV. Various modalities of treatments are available, ranging from conservative therapy to surgery [1,2]. Compression stockings, foam sclerotherapy, radiofrequency ablation, and endovenous laser therapy are available for managing VV [3-10]. However, the gold standard treatment of VV still is surgical flush ligation of the great saphenous vein and stripping incompetent veins with perforator ligation [11,12].

Assessing patients' quality of life has received increasing attention, mainly because questions have been raised regarding the direct benefits of the treatment provided. Hence, clinical outcome and quality of life (QoL) after chronic venous disease (CVD) treatment must be measured. Patient-completed tools mainly focus on general morbidity or disease-specific morbidity. Physician-driven tools are based on their observations, classifying the disease, and evaluating clinically relevant changes over time. A revised venous clinical severity score (rVCSS) documents and evaluates CVD progression [13,14]. Chronic venous insufficiency quality of life questionnaire (CIVIQ)-14 score is widely used to assess disease-specific QoL in CVD [15,16].

Several studies were performed to evaluate clinical outcomes or QoL separately [17,18]. The effect of the surgical procedure on the clinical outcome and QoL of a patient with CVD has been studied either from a clinician's perspective or via questionnaires from a patient's perspective. However, it will be helpful if the two scoring systems are compared and assessed regarding their correlation and agreement. There are no studies in the past where rVCSS and CIVIQ-14 scores were used together to determine the clinical outcomes and QoL in patients of CVD undergoing surgical treatment. In the present study, rVCSS and CIVIQ-14 are evaluated together to demonstrate a positive effect on clinical outcomes and QoL in patients of VV undergoing Trendelenburg's operation with subfascial ligation of perforators [19].

## Materials and methods

The study was conducted in the Department of Surgery, Jawaharlal Institute of Postgraduate Medical Education and Research (JIPMER), Puducherry, India, from September 2019 to March 2020. It was approved by the institute ethics committee (JIP/IEC/2019/257). The study was a prospective observational cohort study of a single group of patients undergoing Trendelenburg’s surgery with subfascial perforator ligation for VV. All patients of both genders above 18 years of age, with great saphenous VV planned for Trendelenburg’s surgery with subfascial perforator ligation, were included in the study. Patients with associated short saphenous VV, pregnancy, unfit for surgery, uncontrolled medical conditions with leg edema, and those with a previous VV surgery or intervention were excluded from the study.

The sample size was estimated using the sample size formula for comparing more than two dependent means. Over time, the minimum expected mean difference in the rVCSS and CIVIQ-14 score was two, with a standard deviation (SD) of five [20]. The sample size was estimated at a 5% level of significance and 90% power with an intraclass correlation of 0.90. The sample size was further inflated with an expected dropout of 20%. The final sample size estimated for the study was 52. A convenient sampling technique was followed.

**Figure 1:**
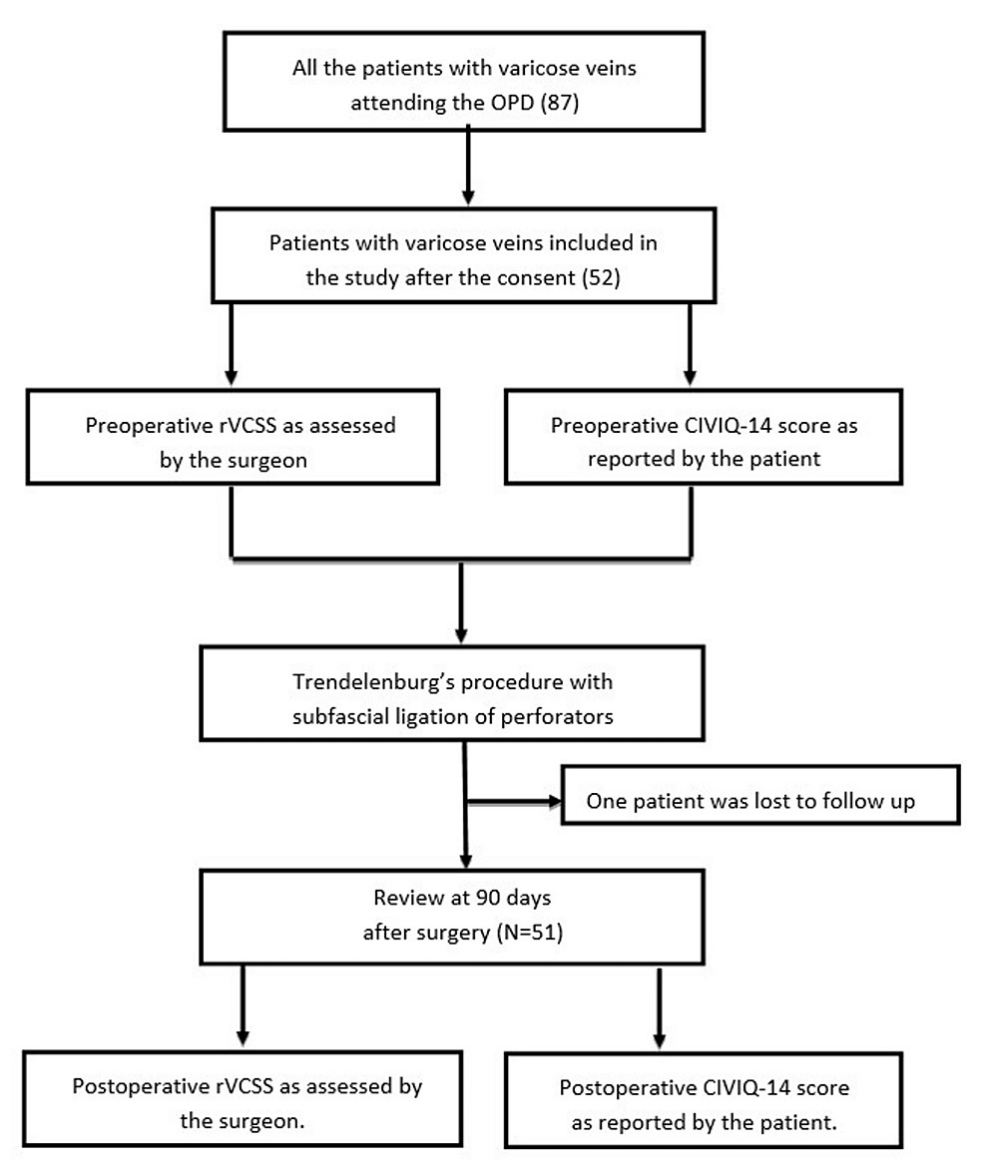
Consolidated Standards of Reporting Trials (CONSORT) diagram and allocation. rVCSS: revised venous clinical severity score; CIVIQ-14: chronic venous insufficiency quality of life questionnaire-14; OPD: Outpatient department; N: Total number of patients.

Informed consent was obtained from patients. VV disease was classified as per clinical, etiological, anatomical, and pathophysiological (CEAP) classification, and the patient underwent Trendelenburg’s surgery with subfascial perforator ligation. On the day before surgery, the surgeon assessed the clinical severity of the disease using rVCSS. The preoperative QoL was evaluated using the CIVIQ-14 questionnaire. The patient filled out the questionnaire the day before the surgery. All patients underwent standard Trendelenburg's surgery with subfascial ligation of leg perforators. All patients had the same perioperative care. After the surgery, patients were discharged and reviewed in the outpatient department (OPD) after a week. Patients were reviewed again after 90 days during their OPD follow-up, and the surgeon assessed the postoperative clinical severity of the disease using rVCSS. Also, the post-operative QoL was evaluated using the CIVIQ-14 questionnaire, which the patient filled out (Figure 1).

The data was collected in individual patient proforma and was entered systematically in a Microsoft Excel sheet (Redmond, WA, USA). Statistical analysis determined the preoperative and postoperative rVCSS pattern and CIVIQ-14 scores. The correlation between both scores was analyzed using Statistical Package for the Social Sciences (SPSS) 19.0 software (IBM Corp., Armonk, NY, USA). The data on categorical variables, such as gender, and clinical characteristics were expressed as frequency and percentages. The normal distribution of data was tested using Kolmogorov-Smirnov (K-S) test. The data such as age, rVCSS, and CIVIQ-14 score were expressed as mean with SD and median with range.

The change in the rVCSS and CIVIQ-14 scores were compared over time using one-way repeated measures of analysis of variance (ANOVA). The linear relationship between rVCSS and CIVIQ-14 score was explained using Spearman’s co-relational analysis. The changes in rVCSS and CIVIQ-14 scores over time between different clinical and socio-demographic characteristics were explained using two-way repeated measures of ANOVA. All statistical analysis was carried out at a 5% level of significance.

## Results

A total of 87 patients with varicose veins were screened, and 52 patients fulfilling the inclusion criteria were included in the study. One patient was lost to follow-up. Of the 51 patients, 38 (74.5%) were males and 13 (25.5%) were females. The mean (±SD) age among the study participants was 47.82 (±12.49) years. The youngest patient was 24 years of age, and the eldest patient was 77 years. Maximum patients belonged to 51 years to 60 years of age, and only one patient belonged to 71 years to 80 years of age (Table 1).

**Table 1:** The distribution of age among the patients with varicose veins. N: Number of patients.

Age range (years)	Number of patients (N)=51
20-30	6
31-40	9
41-50	13
51-60	17
61-70	5
71-80	1

**Figure 2:**
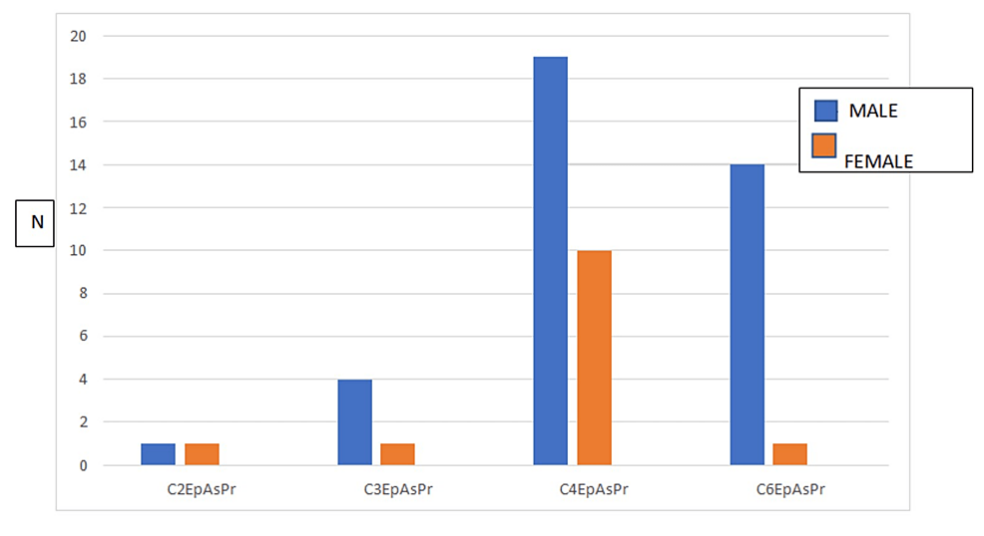
Gender distribution among the various Clinical, Etiological, Anatomical, and Pathophysiological (CEAP) classes in patients with varicose veins. N: Number of patients.

The gender distribution among various CEAP classes had an equal distribution of males and females among C2 class. There was a proportionate increase in the male patients compared to females in C3 class onwards (Figure 2).

We studied the preoperative and postoperative rVCSS among the different CEAP classes. There was a significant difference in the preoperative and postoperative mean of the C3, C4, and C6 stages of the disease (p-value = <0.01). The difference between the scores in the C2 stage could not be measured as only two patients were present. It was observed that there was a significant improvement in the clinical outcomes measured by the rVCSS. We compared the mean preoperative and postoperative CIVIQ-14 scores among the different CEAP classes. There was a significant difference in the mean preoperative and postoperative scores in C3, C4, and C6 (p-value = <0.01). The difference between the scores in the C2 stage could not be measured as only two patients were present in this group (Table 2).

**Table 2:** The distribution of preoperative and postoperative revised venous clinical severity score (rVCSS) and chronic venous insufficiency quality of life questionnaire (CIVIQ)-14 among the different classes. ^$^Data presented as mean ± standard deviation; ^#^Two-way ANOVA test; *Test of significance was not applied as the frequency was less; CEAP: Clinical, Etiological, Anatomical, and Pathophysiological; rVCSS: revised venous clinical severity score; CIVIQ-14: chronic venous insufficiency quality of life questionnaire 14; NA: Not applicable.

CEAP Stage	Preoperative rVCSS^$^	Postoperative rVCSS^$^	p value^#^	Preoperative CIVIQ-14 score^$^	Postoperative CIVIQ-14 score^$^	p-value^#^
C2EpAsPr*	6 ± 2.82	6 ± 4.24	NA	28.50 ± 9.19	14.50 ± 0.70	NA
C3EpAsPr	9.20 ± 1.30	4 ± 1.87	<0.01	32 ± 4.30	15.80 ± 2.68	<0.01
C4EpAsPr	10.97 ± 2.19	5.62 ± 2.32	<0.01	38.21 ± 9.68	19.66 ± 4.18	<0.01
C6EpAsPr	16.87 ± 3.81	7.40 ± 3.18	<0.01	42.80 ± 9.42	21 ± 6.25	<0.01

The comparison of the preoperative and postoperative rVCSS and CIVIQ-14 scores in patients with varicose veins showed a significant difference in preoperative and postoperative rVCSS. There was a significant difference in the mean (±SD) preoperative CIVIQ-14 score and the mean (±SD) postoperative CIVIQ-14 score (p-value = <0.01). Thus, it was observed that there was a significant improvement in the overall (irrespective of the disease stage) clinical outcomes and the QoL measured by rVCSS and CIVIQ-14 scores, respectively (Table 3).

**Table 3:** The comparison of the preoperative and postoperative revised venous clinical severity score (rVCSS) and chronic venous insufficiency quality of life questionnaire 14 (CIVIQ-14) scores in the patients with varicose veins. *Wilcoxon Signed Ranks test; ^#^paired students t test; IQR: Interquartile range; SD: standard deviation; rVCSS: revised venous clinical severity score; CIVIQ-14: chronic venous insufficiency quality of life questionnaire 14

Score	Preoperative score	Postoperative score	p-value
rVCSS [median (IQR) ]	11 (10,14)	6 (4,7)	<0.01*
CIVIQ-14 score [mean± SD]	38.57 ± 9.71	19.47 ± 4.90	<0.01^#^

The correlation coefficient between the CIVIQ-14 preoperative score and the rVCSS preoperative score is 0.26, a weak monotonic relationship. However, it was insignificant as the p-value was 0.58. The correlation coefficient between the CIVIQ-14 postoperative score and the rVCSS postoperative score is 0.42, a moderate monotonic relationship and significant (p-value = <0.01). Thus, there was no significant correlation between the preoperative stage of the disease and the preoperative QoL. In contrast, there was a significant correlation between the postoperative stage of the disease and the postoperative QoL measured by rVCSS and CIVIQ-14 score, respectively (Table 4).

**Table 4:** Correlation of preoperative and postoperative revised venous clinical severity score (rVCSS) and chronic venous insufficiency quality of life questionnaire-14 (CIVIQ-14) score. *spearman’s rank correlation test; ^#^spearman’s rho; rVCSS: revised venous clinical severity score; CIVIQ-14: chronic venous insufficiency quality of life questionnaire-14; NA: not applicable.

Statistical test	CIVIQ-14 (Preoperative score)	rVCSS (preoperative score)	CIVIQ-14 (Postoperative score)	rVCSS (postoperative score)
Correlation coefficient	1	0.26^#^	1	0.42^#^
p-value	NA	0.58*	NA	<0.01*

## Discussion

Surgery for VV is the most common management mode of lower limb VV, especially in centers where radiofrequency or laser ablation facilities are unavailable. VV have been classified traditionally using CEAP classification [20,21]. There exist several recent studies on minimally invasive procedures to treat VV. However, the open surgical method is still practiced in most surgical centers in India. Our study evaluated the postoperative improvement of clinical outcomes evaluated by rVCSS and the QoL assessed by CIVIQ-14 score three months following Trendelenburg’s surgery and subfascial ligation of perforators. We assessed the outcomes of surgery in 51 patients. There was a significant improvement in the overall clinical outcomes and the QoL after the surgery.

In our study, most of the patients were male. Patients with high severity of the disease were also male compared to females. These differences could be because most patients were of lower socioeconomic status, and females of lower socioeconomic status were ignorant about the symptoms and were not willing for surgery.

Kurz et al. conducted a multicenter cross-sectional study to evaluate the QoL in patients with VV, using Short-Form Health Survey-36 (SF-36) and Venous Insufficiency Epidemiological and Economic Study on Quality of Life/Symptoms (VEINES-QOL/SYM) scale [22]. They included 1054 patients with VV. They were staged using CEAP grading, and 34.8% of patients had C2 disease, 11.9% had C3 disease, 40.9% had C4 disease, 9.5% had C5 disease, and 2.9% had C6 disease. They reported a statistically significant difference in all the scores (p-value = <0.01) among all the CEAP classes compared to the normal population without VV [22]. In the present study, 3.9% of patients had C2 disease, 9.8% had C3, 56.9% had C4, and 29.4% had C6 disease. Compared to this study, most patients in our study had stage C4 disease. There was a significant improvement in the postoperative clinical outcomes and QoL (p-value = <0.01).

Radak et al. used the numeric rating scale to study the relationship between pain and CEAP C categories of CVD [15]. This scale ranged from zero to five units and assessed pain intensity. Almost 90.5% of the patients with venous insufficiency reported pain in the lower limbs as their complaint. They found that the higher the clinical stage, the more the occurrence of pain in those patients (p-value = <0.01) [15]. In the present study, there was an increase in the rVCSS and CIVIQ-14 scores as the C class of the disease was increasing. This increase might be due to the increased severity of the disease as the C class rises.

Starves et al. conducted an observational study with 45 patients who underwent VV surgery [23]. They assessed and compared venous clinical severity score (VCSS) and CEAP clinical classes before surgery, six weeks, and six months after surgery. Pre-procedure mean VCSS was 10.7, and the post-procedure VCSS was 7.6, which is statistically significant (p-value = <0.01). It was found that a correlation was present between all the severity scores. Especially to mention a significant correlation between CEAP clinical stage and VCSS [23]. Bountouroglou et al. compared the CEAP class, VCSS, and Aberdeen vein questionnaire (AVQ) between ultrasound-guided foam sclerotherapy combined with saphenofemoral ligation compared to surgical treatment of VV [24]. There was a significant decrease in scoring in both groups. There was a 46% decrease at three months in the sclerotherapy group and 40% in the conventional surgery group [24]. In the present study, the median preoperative rVCSS was 11. Surgery in the patients with VV significantly improved QoL as the postoperative score significantly reduced, with a median score of 6 (p-value = <0.01). There is also a significant difference in the preoperative and postoperative rVCSS in the different CEAP stages of the disease (p-value = <0.01), suggesting improvement in the disease stage, like in previous studies.

Surgery in CVD has a significant improvement in the QoL. Baker et al. studied the QoL in 150 patients undergoing surgery for VV, using the SF-36 health assessment questionnaire done before surgery, at one month and six months postoperatively [25]. They found that all dimensions improved, besides social function and health perception, within six months post-surgery, compared to preoperative values (p-value = <0.01). There was symptomatic improvement within one month after surgery, and further improvement was present six months post-surgery (p-value = <0.05) [25]. In the present study, the mean preoperative CIVIQ-14 score was 38. There was a drastic improvement in the postoperative score as the mean postoperative CIVIQ-14 score reduced to 19, which is about a 50% reduction and is significant (p-value = <0.01). Our results are like previous studies suggesting there is improvement in QoL in patients who underwent surgery.

Launois et al. studied the QoL in patients with C0 to C4 CEAP disease using the chronic venous insufficiency questionnaire-20 (CIVIQ-20) questionnaire in 3956 patients [26]. They reported a statistically significant difference in the QoL and CEAP stages of the disease (p-value = 0.0001). Radak et al. studied the QoL in patients with CVD using the CIVIQ-14 questionnaire [15]. QoL was significantly reduced in CEAP class C0 to C6 [15]. In our study, there is an increase in the CIVIQ-14 score with the higher CEAP stage, which signifies a poor QoL with a severe disease stage. The present study shows no significant correlation between the preoperative rVCSS and CIVIQ-14 score, showing that the disease significantly impacts the QoL (p-value = 0.58). However, it is of a lower clinical stage. There is a significant correlation between the clinician-graded severity and the patient's QoL postoperatively, but it is not clinically relevant. Our study also observed that patients perceive the same quality improvement in QoL with different (C3, C4, C6) clinical grades of disease with surgical intervention (p-value = 0.58).

There are a few limitations of our study. The study was observational, and there was no control group. Due to the low sample size of the study, the distribution of patients in different CEAP classes could have been more stable. There were a significantly smaller number of patients (only two) in the C2 class of VV and no patients under the clinical stage C5 of the disease. Hence, the effect of the surgery could not be assessed statistically in the C2 and C5 clinical stages of the disease. It was impossible to measure the difference between preoperative and postoperative rVCSS and CIVIQ-14 scores among these stages of the disease. Hence, it is not easy to extrapolate the findings to the general population. Also, because of the less sample size, there is a gender disparity, where male participants were almost twice as females. Recall bias could be present as it was a questionnaire format, and few patients needed to be literate; this could have influenced the scores.

## Conclusions

In the present study, all patients undergoing Trendelenburg’s surgery and subfascial ligation of perforators for varicose veins significantly improved the clinical severity and quality of life. There was significant improvement among the overall rVCSS and CIVIQ-14 score after surgery among the different CEAP clinical classes. Each of the parameters among both scores showed improvement postoperatively. There was no preoperative correlation between the rVCSS and CIVIQ-14 score. However, there was a significant correlation between the postoperative rVCSS and CIVIQ-14 score.

## References

[1] Araujo DN, Ribeiro CT, Maciel AC, Bruno SS, Fregonezi GA, Dias FA (2023). Physical exercise for the treatment of non-ulcerated chronic venous insufficiency. Cochrane Database Syst Rev.

[2] Takase S, Pascarella L, Bergan JJ, Schmid-Schönbein GW (2004). Hypertension-induced venous valve remodeling. J Vasc Surg.

[3] Raju S, Hollis K, Neglen P (2007). Use of compression stockings in chronic venous disease: patient compliance and efficacy. Ann Vasc Surg.

[4] Bergan J (2000). Non-elastic compression: an alternative in the management of chronic venous insufficiency. J WOCN.

[5] Tessari L, Cavezzi A, Frullini A (2001). Preliminary experience with a new sclerosing foam in the treatment of varicose veins. Dermatol Surg.

[6] Hamel-Desnos C, Desnos P, Wollmann JC, Ouvry P, Mako S, Allaert FA (2003). Evaluation of the efficacy of polidocanol in the form of foam compared with liquid form in sclerotherapy of the greater saphenous vein: initial results. Dermatol Surg.

[7] Min RJ, Zimmet SE, Isaacs MN, Forrestal MD (2001). Endovenouslaser treatment of the incompetent greater saphenous vein. J Vasc Interv Radiol.

[8] Lurie F, Creton D, Eklof B (2003). Prospective randomized study of endovenous radiofrequency obliteration (closure procedure) versus ligation and stripping in a selected patient population (EVOLVeS Study). J Vasc Surg.

[9] van den Bos R, Arends L, Kockaert M, Neumann M, Nijsten T (2009). Endovenous therapies of lower extremity varicosities: a meta-analysis. J Vasc Surg.

[10] Venermo M, Saarinen J, Eskelinen E (2016). Randomized clinical trial comparing surgery, endovenous laser ablation and ultrasound-guided foam sclerotherapy for the treatment of great saphenous varicose veins. Br J Surg.

[11] Ortega MA, Fraile-Martínez O, García-Montero C (2021). Tissue remodelling and increased DNA damage in patients with incompetent valves in chronic venous insufficiency. J Cell Mol Med.

[12] Hernández-Morera P, Castaño-González I, Travieso-González CM, Mompeó-Corredera B, Ortega-Santana F (2016). Quantification and statistical analysis methods for vessel wall components from stained images with masson's trichrome. PLoS One.

[13] Gao RD, Qian SY, Wang HH, Liu YS, Ren SY (2022). Strategies and challenges in treatment of varicose veins and venous insufficiency. World J Clin Cases.

[14] Silverberg J, Jackson JM, Kirsner RS (2023). Narrative review of the pathogenesis of stasis dermatitis: an inflammatory skin manifestation of venous hypertension. Dermatol Ther (Heidelb).

[15] Radak DJ, Vlajinac HD, Marinković JM, Maksimović MŽ, Maksimović ZV (2013). Quality of life in chronic venous disease patients measured by short Chronic Venous Disease Quality of Life Questionnaire (CIVIQ-14) in Serbia. J Vasc Surg.

[16] Le Moine JG, Fiestas-Navarrete L, Katumba K, Launois R (2016). Psychometric validation of the 14 items ChronIc Venous Insufficiency Quality of Life Questionnaire (CIVIQ-14): confirmatory factor analysis. Eur J Vasc Endovasc Surg.

[17] Pascarella L, Schönbein GW, Bergan JJ (2005). Microcirculation and venous ulcers: a review. Ann Vasc Surg.

[18] Raffetto JD (2014). Which dressings reduce inflammation and improve venous leg ulcer healing. Phlebology.

[19] Linton RR (1938). The communicating veins of the lower leg and the operative technic for their ligation. Ann Surg.

[20] Eklöf B, Rutherford RB, Bergan JJ (2004). Revision of the CEAP classification for chronic venous disorders: consensus statement. J Vasc Surg.

[21] Lurie F, Passman M, Meisner M (2020). The 2020 update of the CEAP classification system and reporting standards. J Vasc Surg Venous Lymphat Disord.

[22] Kurz X, Lamping DL, Kahn SR, Baccaglini U, Zuccarelli F, Spreafico G, Abenhaim L (2001). Do varicose veins affect quality of life? Results of an international population-based study. J Vasc Surg.

[23] Kakkos SK, Rivera MA, Matsagas MI, Lazarides MK, Robless P, Belcaro G, Geroulakos G (2003). Validation of the new venous severity scoring system in varicose vein surgery. J Vasc Surg.

[24] Bountouroglou DG, Azzam M, Kakkos SK, Pathmarajah M, Young P, Geroulakos G (2006). Ultrasound-guided foam sclerotherapy combined with sapheno-femoral ligation compared to surgical treatment of varicose veins: early results of a randomised controlled trial. Eur J Vasc Endovasc Surg.

[25] Baker DM, Turnbull NB, Pearson JCG, Makin GS (1995). How successful is varicose vein surgery? A patient outcome study following varicose vein surgery using the SF-36 health assessment questionnaire. Eur J Vasc Endovasc Surg.

[26] Launois R, Mansilha A, Jantet G (2010). International psychometric validation of the Chronic Venous Disease quality of life Questionnaire (CIVIQ-20). Eur J Vasc Endovasc Surg.

